# Time series gene expression profiling and temporal regulatory pathway analysis of BMP6 induced osteoblast differentiation and mineralization

**DOI:** 10.1186/1752-0509-5-82

**Published:** 2011-05-23

**Authors:** Weijun Luo, Michael S Friedman, Kurt D Hankenson, Peter J Woolf

**Affiliations:** 1 Department of Biomedical Engineering, University of Michigan, Ann Arbor, MI 48109, USA; 2 Cold Spring Harbor Laboratory, Cold Spring Harbor, NY 11724, USA; 3 Thermogenesis Corporation, Rancho Cordova, CA 95742, USA; 4 Department of Animal Biology, University of Pennsylvania, Philadelphia, PA 19104, USA; 5 Department of Chemical Engineering, University of Michigan, Ann Arbor, MI 48109, USA; 6 Bioinformatics Program, University of Michigan, Ann Arbor, MI 48109, USA

## Abstract

**Background:**

BMP6 mediated osteoblast differentiation plays a key role in skeletal development and bone disease. Unfortunately, the signaling pathways regulated by BMP6 are largely uncharacterized due to both a lack of data and the complexity of the response.

**Results:**

To better characterize the signaling pathways responsive to BMP6, we conducted a time series microarray study to track BMP6 induced osteoblast differentiation and mineralization. These temporal data were analyzed using a customized gene set analysis approach to identify temporally coherent sets of genes that act downstream of BMP6. Our analysis identified BMP6 regulation of previously reported pathways, such as the TGF-beta pathway. We also identified previously unknown connections between BMP6 and pathways such as Notch signaling and the MYB and BAF57 regulatory modules. In addition, we identify a super-network of pathways that are sequentially activated following BMP6 induction.

**Conclusion:**

In this work, we carried out a microarray-based temporal regulatory pathway analysis of BMP6 induced osteoblast differentiation and mineralization using GAGE method. This novel temporal analysis is more informative and powerful than the classical static pathway analysis in that: (1) it captures the interconnections between signaling pathways or functional modules and demonstrates the even higher level organization of molecular biological systems; (2) it describes the temporal perturbation patterns of each pathway or module and their dynamic roles in osteoblast differentiation. The same set of experimental and computational strategies employed in our work could be useful for studying other complex biological processes.

## Background

Osteoblasts are responsible for bone matrix production and mineralization [1]. In concert with osteoclasts, osteoblasts coordinate bone remodeling, a physiologic process by which bone mass is maintained [1]. Osteoblasts arise from mesenchymal stem cells (MSC) [1]. However, the mechanism controlling this differentiation process is not well understood.

MSC differentiation to osteoblasts can be induced both *in vivo *and *in vitro *by soluble factors in the bone morphogenetic protein family (BMP) [2,3]. Among the BMPs, BMP2, 4, 6 and 7 are the best characterized osetogenic factors [4]. Our previous work [5] has shown that: (1) human MSC produce BMP6 in defined, serum-free conditions, (2) BMP6 is up-regulated under mild osteogenic stimulus (dexamethasone), (3) exogenous BMP6 potently induces osteoblast differentiation, but responses to BMP2, 4, or 7 are inconsistent and require higher doses, (4) exogenous BMP6 induces the expression or up-regulation of a set of osteoblast-related genes in human MSC, and (5) 24 hour treatment with BMP6 induce high levels of osteoblast gene expression and cause mineralization. These results established BMP6 as an endogenous regulator of human osteoblast differentiation [5].

Unfortunately, BMP6 signaling largely remains uncharacterized. Significant efforts, particularly a series of high throughput microarray studies [6-12] have been undertaken to uncover BMP (including BMP6) responsive genes, transcriptional programs, and their roles in osteoblast development. However, an integrated understanding of the regulatory mechanisms for osteoblast differentiation and mineralization has not been achieved. In particular, two remaining questions include: (1) what pathways and gene groups are responsible for MSC differentiation to osteoblasts in response to BMP6 stimulation? (2) what is the sequence of pathway activation events in response to BMP6 during the osteogenic induction?

To answer these two questions, we conducted a time series microarray study on BMP6 osteogenic induction and a comprehensive pathway analysis on the temporal data. We met special challenges at both experiment and data analysis levels.

**Figure 1:**
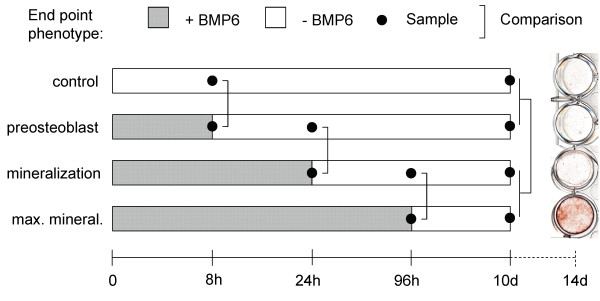
**Design for the microarray study on BMP6 induced osteoblast differentiation**. Human MSC cells were pre-cultured for 4 days and subsequently treated with BMP6 for 0 hours, 8 hours, 24 hours, and 96 hours. These four time points correspond to four phenotypic groups, of control, preosteoblast (no mineralization), (sub-maximal) mineralization, and maximal mineralization at 14 days (18 days in total). Cells were harvested at 8 hours, 24 hours, 96 hours and 10 days for microarray profiling. Mineralization level was quantified at 14 days by Alizarin Red S staining (right column). GAGE was applied to infer the most differentially expressed pathways or gene sets between the matched samples with or without BMP6 at different time points. At time points corresponding to 8, 24 and 96 hours, GAGE compares between only two sample conditions. At the 10 day time point, GAGE compares between two mineralized conditions versus two non-mineralized conditions.

At experiment level, we considered two major issues. First, what rate to sample the time series? The time intervals should be short enough to capture the dynamics and continuity, but long enough to show phenotypically significant changes. We determined time intervals based on the minimal BMP6 treatment durations needed for significant phenotypic changes, including the expression of osteoblast markers and formation of mineralized extracellular matrix (Figure 1). Second, given that osteoblast induction is a temporal process with accumulative effects and gradual changes, how to dissect out the net effect of BMP6 at different phenotypic stages? We employed a BMP6 addition-and-withdrawal scheme (Figure 1).

At data analysis level, we developed a novel temporal pathway analysis procedure. Gene set analysis (GSA) is a well established strategy to identify pathways or gene sets associated with a particular phenotype or condition [13-16]. Temporary analysis was done on biological processes previously [17]. However, traditional GSA methods do not directly apply without externally specified selection criteria for temporal changes. Methods like GSEA [13] accept continuous phenotype labels hence can correlate gene set changes to time or pre-defined temporal patterns. To our knowledge, these methods do not infer temporal pathway level changes without reference patterns. For such temporal pathway analysis to be practical, a method needs to be: (1) applicable to datasets with small or even changing sample size at each time point or condition. (2) both sensitive and selective to capture subtle yet real regulatory signals over time. To meet these challenges, we designed a special analysis procedure (Figure 1) based on our newly developed GAGE (Generally Applicable Gene-set Enrichment) method [18].

Using this joint experimental and computational approach, we identified both the pathways and their temporal responses to BMP6 signaling during osteoblast differentiation and mineralization.

## Results

Following our previous study [5], we explored BMP6 induced human MSC osteoblast gene expression and function. Our preliminary experiments showed that 8 hours BMP6 exposure was sufficient to induce expression of osteoblast differentiation marker genes in human MSCs. A minimum of 24 hours exposure was required to form mineralized matrix at 14 days after the initiation of BMP treatment. A maximal mineralization response was observed upon 96 hours of BMP6 treatment.

We designed a microarray study to explore the regulatory mechanisms underneath these phenotypic changes at different stages of human MSC osteoblast differentiation (Figure 1). Our recently developed GAGE method [18] was applied to infer the significantly perturbed KEGG (Kyoto Encyclopedia of Genes and Genomes) pathways, GO (Gene Ontology) term groups, and experimentally derived gene sets (experimental sets for short) by BMP6 treatment at different times along the induction process (Figure 1). We examined these significant gene sets in details below.

### Significantly perturbed KEGG pathways during BMP6 osteogenic induction

**Figure 2:**
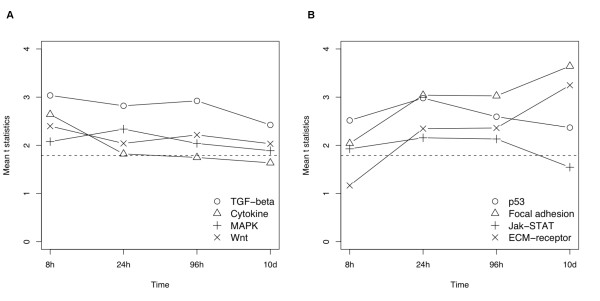
**The expression perturbation patterns induced by BMP6 treatment in eight significant KEGG pathways**. These pathways are consistently significantly differentially expressed or near so based on GAGE. The mean t-statistics from multiple one-on-one comparisons between the two sample conditions is used as overall perturbation magnitude for each pathway. Perturbation magnitude here is defined as the absolute value of both positive and negative gene expression changes without considering the actual perturbation direction. The dashed line indicates a t-statistic of 1.79, which roughly corresponds to p = 0.01 for 8-96 h or 0.001 for 10 d. Panel (a) and (b) are divided only for visual clarity.

We observed a single set of KEGG pathways that were significantly perturbed at the gene expression level throughout the BMP6 induction process (Figure 2F and Table 1). This result suggests that these regulatory mechanisms are constantly involved at different stages of BMP6 induced osteoblast differentiation and mineralization. To test whether a KEGG pathway are significantly associated with a phenotype or a sample condition, we account for gene expression level perturbation in both directions (both up- and down-regulation), because a pathway commonly includes both positive and negative effectors and local feedback loops to keep the system in balance [18]. Therefore, we refer to a KEGG pathway as significantly perturbed rather than activated or inhibited as a whole.

**Table 1:** Interpretation and validation of the significant KEGG pathways inferred by GAGE.

KEGG pathways	Most perturbed	References	Other evidences
TGF-beta signaling pathway	8h	[5,57]	BMP signal targets IDs, SMAD6-7, DLX5 extremely up

Cytokine-cytokine receptor interaction	8h	[33,58]	IFN target gene sets down (Table 4)

Wnt signaling pathway	8h	[59,60]	

Jak-STAT signaling pathway	24h	[58,61]	STAT1 target genes down

MAPK signaling pathway	24h	[22,23]	--

p53 signaling pathway	24h	[62,63]	--

Focal adhesion	10d	[20,21]	--

ECM-receptor interaction	10d	[64,65]	--

Literature findings and experimental evidence suggest regulatory roles of these KEGG pathways in BMP6 induced osteoblast differentiation and mineralization.

These significant KEGG pathways show different temporal perturbation patterns (Figure 2). The TGF-beta signaling pathway, Cytokine-cytokine receptor interaction, and Wnt signaling pathway are most perturbed at 8h. The Jak-STAT signaling pathway, MAPK signaling pathway and p53 signaling pathway are most perturbed at 24h. Focal adhesion and ECM-receptor interaction are most perturbed at 10d.

**Figure 3:**
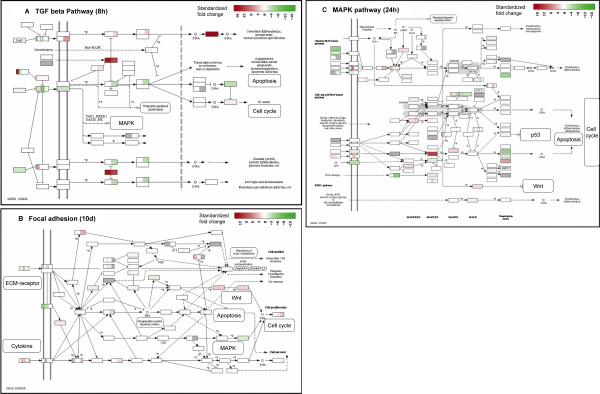
**Gene expression fold changes induced by BMP6 in three representative significant KEGG pathways**. Each pathway shown is at its most perturbed time point: (a) TGF-beta signaling pathway at 8h; (b) Focal adhesion at 10d; (c) MAPK signaling pathway at 24h. Gene expression level fold changes are standardized over the standard deviation of fold changes for all genes. The standardized fold changes are visualized by using KEGGanim web tool [56]. Note that one KEGG node may correspond to multiple closely related genes with the same function. Relevant other pathways are magnified locally for better view. Gene names are intentionally omitted by KEGGanim for clear view of the gene expression changes in pseudo-color.

We explored the pattern of differential gene expression induced by BMP6 treatment in three representative pathways in detail (Figure 3 a-c and Additional file 1,2,3,4,5). First, TGF-beta signaling pathway is the top significant pathway (compared to other pathways) at 8h and is most perturbed (compared to itself at other time points) also at 8h. Therefore, this pathway is likely triggered directly by BMP6 treatment, and could be the signal initiating the osteoblast differentiation. This role of TGF-beta signaling is consistent with previous work [19] and the common sense: BMP6 as a canonical BMP triggers canonical BMP signaling, which is one major branch of TGF-beta signaling pathway. Second, focal adhesion is the most significant pathway (compared to other pathways) after 24h and is most perturbed (compared to itself at other time points) at 10 days. This pathway is likely the convergence point of the regulatory signals, and it is associated with late stage osteoblast differentiation and mineralization. These results are consistent with the role of focal adhesion inferred from experimental work in the literature [20,21]. Third, the MAPK signaling pathway is the most perturbed at 24h or the middle stage of differentiation, which suggests that this pathway is likely an intermediate step during the BMP6 induced signal relay process at gene expression level. Indeed, MAPKs have been reported to mediate BMP effect during osteoblast differentiation [22,23]. The temporal perturbation patterns in other significant pathways suggest their functions in the BMP6 induction process, which are supported by both observations in the literature and evidence from the expression data (Table 1). The temporal roles assigned to the pathways are relative, because all these pathways are important throughout BMP6 induced osteoblast differentiation and mineralization (Figure 2).

**Figure 4:**
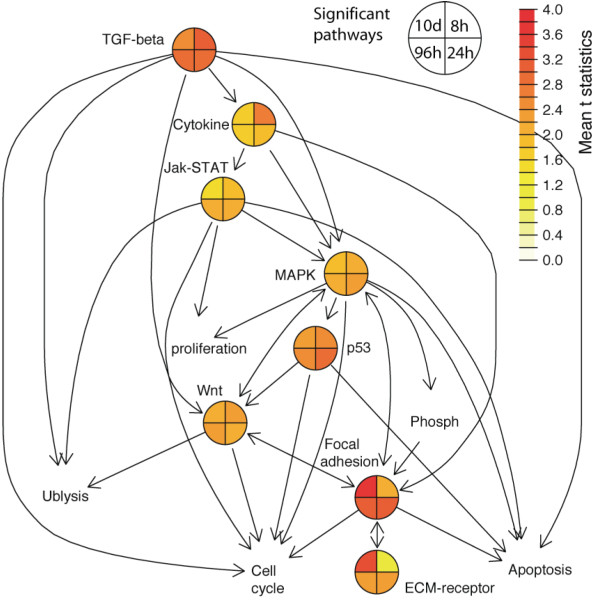
**An integrated network of the significant KEGG pathways with their temporal perturbation patterns**. Significant KEGG pathways (colored pie charts) or closely related other pathways (text only) are connected by arrows as indicated in the KEGG database. The mean t-statistics from multiple one-on-one comparisons at four different time points are plotted in pseudo heat color in the pie nodes as the average perturbation magnitude for significant KEGG pathways. Full names for some abbreviated KEGG pathways are: Cytokine-cytokine receptor interaction (Cytokine), Ubiquitin mediated proteolysis (Ublysis), Phosphatidylinositol signaling system (phosph).

Interestingly, the significant KEGG pathways are not distinct but rather act as an integrated super regulatory system. They interconnect to each other as shown on KEGG pathway graphs (Figure 3a-c). For example, the TGF-beta pathway triggers the MAPK signaling pathway (Figure 3a), whereas MAPK signaling pathway connects to focal adhesion (Figure 3b). These pathways also share common downstream response processes including apoptosis, cell cycle etc (Figure 3a-c). Using the top KEGG pathways inferred by GAGE [18], gene expression data and connections between pathways from LinkDB module of KEGG databases [24], we created an integrated dynamic network view of pathways in Figure 4. The upstream nodes including TGF-beta signaling pathway and cytokine-cytokine receptor interaction are most perturbed at the early stage. Downstream nodes including focal adhesion and ECM-receptor interaction are most perturbed at the late stage. Midstream nodes including MAPK signaling and p53 signaling are most perturbed at the middle stage. These sequential perturbation patterns across interconnected pathways suggest a dynamic transmission process of the regulatory signals induced by BMP6 treatment at the transcriptional level.

The connected significant pathways (Figures 4) often share component genes that are evidently perturbed in expression (Table 2). Particularly, overlaps between significant KEGG pathways (Table 2) are consistent with the connections between pathways (Figure 4): high overlaps (Table 2) almost always suggest direct connections between pathways (Figure 4), and vise versa. Overlapping component genes serve as bridges across these relatively independent functional modules or pathways, hence perturbation in one pathway such as the BMP-TGF-beta signaling pathway can be propagated throughout other relevant pathways. These data suggest that these significant KEGG pathways work together as an integrated regulatory system.

**Table 2:** The overlaps in perturbed member genes between the significant KEGG pathways inferred by GAGE

	TGFb	Cyto	Wnt	MAPK	Jak	p53	Focal	ECM
TGFb	0 (22)	5.6E-20(8)	3.3E-06(2)	4.3E-10(4)	3.1E-04(1)	3.1E-04(1)	2.9E-06(2)	4.1E-05(1)

Cyto	5.6E-20(8)	0(34)	1 (0)	3.4E-11(5)	8.3E-29(11)	1 (0)	6.4E-10(4)	1 (0)

Wnt	3.3E-06(2)	1 (0)	0 (24)	6.9E-10(4)	2.9E-06(2)	3.5E-09(3)	2.3E-08(3)	1 (0)

MAPK	4.3E-10(4)	3.4E-11(5)	6.9E-10(4)	0 (33)	7.0E-04(1)	2.7E-06(2)	5.5E-10(4)	1 (0)

Jak	3.1E-04(1)	8.3E-29(11)	2.9E-06(2)	7.0E-04(1)	0 (21)	1.4E-04(1)	3.4E-04(1)	1 (0)

p53	3.1E-04(1)	1 (0)	3.5E-09(3)	2.7E-06(2)	1.4E-04(1)	0 (15)	3.0E-09(3)	1.9E-05(1)

Focal	2.9E-06(2)	6.4E-10(4)	2.3E-08(3)	5.5E-10(4)	3.4E-04(1)	3.0E-09(3)	0 (23)	2.1E-24(7)

ECM	4.1E-05(1)	1 (0)	1 (0)	1 (0)	1 (0)	1.9E-05(1)	2.1E-24(7)	0 (8)

For each cell in the table, in the parenthesis is the number of perturbed member genes overlapping between the significant KEGG pathways; outside is the significance of this overlap between KEGG pathways. Details of overlap analysis are given in Methods section. Full names for these KEGG pathways are: TGF-beta signaling pathway (TGFb), Cytokine-cytokine receptor interaction (Cyto), Wnt signaling pathway (Wnt), MAPK signaling pathway (MAPK), Jak-STAT signaling pathway (Jak), p53 signaling pathway (p53), Focal adhesion (Focal), ECM-receptor interaction (ECM).

### Significantly perturbed GO term gene sets during BMP6 osteogenic induction

**Figure 5:**
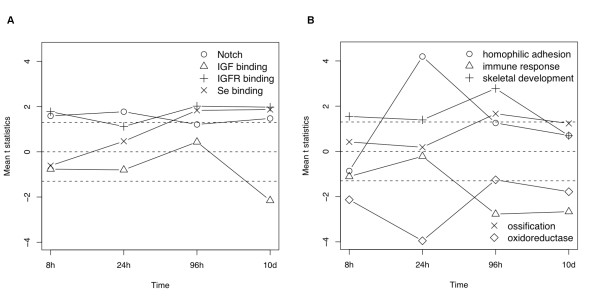
**The expression perturbation patterns induced by BMP6 treatment in nine significant GO term gene sets**. Each of these GO term gene sets is significant in at least one time point based on GAGE. (a) GO:0007219 Notch signaling pathway, GO:0005520 insulin-like growth factor binding, GO:0005159 insulin-like growth factor receptor binding, GO:0008430 selenium binding, (b) GO:0007156 homophilic cell adhesion, GO:0006955 immune response, GO:0001501 skeletal development, GO:0001503 ossification, GO:0016491 oxidoreductase activity. The mean t-statistic from multiple one-on-one comparisons between the two sample conditions is used as a measure of the overall perturbation for each GO term. Different from KEGG pathways, perturbation direction is considered here. The dashed lines mark t = +/-1.30, which roughly correspond to p = 0.05 for 8-96 h or 0.01 for 10 d.

In contrast to the top KEGG pathways, the top significant GO term gene sets change with time (Figure 5 and Table 3). Most relevant GO term gene sets are significantly up or down regulated only for part of the induction process, except for the Notch signaling pathway and insulin-like growth factor receptor binding. In contrast to the KEGG pathways, where we test for two-directional perturbations, we test for one-directional changes in GO term gene sets as a complementary analysis to the KEGG pathway analysis above. More details are described in the Methods section. Additional file 6 includes comprehensive lists of significant KEGG pathways and GO groups at different time points.

**Table 3:** Interpretation and validation information of the significant GO term gene sets inferred by GAGE

GO terms	Perturbation	References	Other evidence
Notch signaling pathway	8h-10d up	[11,25]	--

insulin-like growth factor receptor binding	8h-10d up	[26,27]	--

insulin-like growth factor binding	10d down	[28-31]	--

homophilic cell adhesion	24h-96h up	[34]	--

immune response	96h-10d down	[32,33]	--

skeletal development	8h-96h up	--	--

ossification	96h-10d up	--	--

oxidoreductase activity	8h-24h, 10d down	[35]	--

selenium binding	96h-10d up	--	--

Temporal perturbation patterns (significantly perturbed time period and the direction), supportive literature and experimental evidence all suggest the regulatory roles of these GO terms in BMP6 induced osteoblast differentiation and mineralization.

The top GO term gene sets suggest novel yet plausible regulatory processes involved in BMP6 induced osteoblast differentiation and mineralization. Some GO terms are self-explaining, including skeletal development and ossification (Figure 5b). Other less evident top GO terms are supported by the literature (Table 3). For example, the notch signaling pathway (Figure 5a) is directly involved in BMP2 induced osteoblast differentiation [11,25], suggesting a possible connection to BMP6 too. Both insulin-like growth factor receptor binding and insulin-like growth factor binding (Figure 5a) suggest that the IGF signal is important for osteoblast differentiation and mineralization. Indeed IGF1 [26] and IGF1R [27] promote mineralization and bone formation, whereas IGFBP 3-6 [28-31] sequesters IGF1 and inhibits osteoblast differentiation and mineralization. Direct connections between immune response (Figure 5b) and bone formation and metabolism have been well appreciated [32,33], which merge into a new research area, osteoimmunology [33]. Genes from homophilic cell adhesion [34] and oxidoreductase activity [35] (Figure 5b) sets play a role in osteoblast differentiation. Selenium (Figure 5a) deficiency is associated with osteoporosis [36], a disease in bone formation and metabolism.

### Significantly perturbed experimentally derived gene sets during BMP6 osteogenic induction

**Figure 6:**
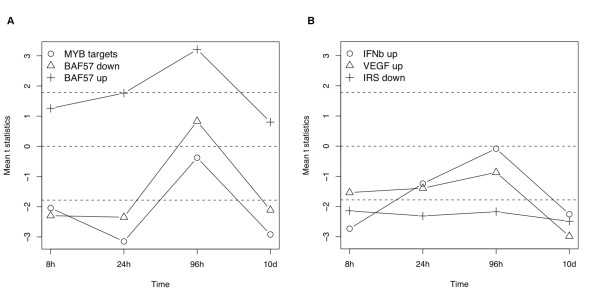
**The expression perturbation patterns induced by BMP6 treatment in six significant experimental sets**. Each of these experimental sets is significantly up or down regulated in at least one time point based on GAGE. (a) MYB targets, BAF57 down, BAF57 up; (b) IFNb up, VEGF up, IRS down. The mean t-statistics from multiple one-on-one comparisons between the two sample conditions is used as overall perturbation for each gene set. Different from KEGG pathways, perturbation direction is considered here. The dashed lines mark t = +/-1.78, which roughly correspond to p = 0.01 for 8-96 h or 0.001 for 10 d.

Our analysis also identified the most significantly up or down-regulated experimental sets during BMP6 induction. Experimental sets are gene groups coregulated or coexpressed in certain chemical or genetic perturbation experiments from the literature. A significant experiment set suggests that their common regulator was perturbed in our experiment too. There are commonly multiple experimental sets describing the same perturbation or mapping to the same regulatory mechanism. A non-redundant subset of the top experimental sets were collected in Table 4 with their perturbation patterns shown in Figure 6. Similar to gene sets based on GO terms but not to those on KEGG pathways, experimental sets were tested for perturbations in only one direction, either up- or down- regulated [18].

**Table 4:** Interpretation and validation information on the significant experimental sets inferred by GAGE

Experimental sets	Significant	References	Other evidences
MYB targets	8h-24h,10d down	[37,38]	Wnt signal (KEGG)

BAF57 down	8h-24h,10d down	[39-42]	BAF57 up

BAF57 up	24h-96h up	[39-42]	BAF57 down

IFNb up	8h,10d down	--	Jak-STAT signal (KEGG)

VEGF up	10d down	[43]	MYB targets

IRS down	8h-10d down	--	IGF signal (GO)

Temporal perturbation patterns, supportive literature works and extra experimental evidences all suggest the regulatory roles of these experimental sets in BMP6 induced osteoblast differentiation and mineralization. Experimental sets came from MSigDB [51] c2 collection, where the original names for these significant gene sets are: Lei_MYB_Regulated_Genes, BAF57_BT549_Dn, BAF57_BT549_Up; (B) Der_IFNB_Up, VEGF_Mmmec_6hrs_Up, IRS_Ko_Adip_Up (down regulated by IRS since up-regulated upon IRS knock down).

MYB transcription factor is identified as a novel regulator for osteoblast differentiation. MYB target gene set was down regulated at 8h, 24h and 10d with no change in MYB expression level. MYB transcriptional activity at protein level can be inhibited through two potential mechanisms following BMP6 treatment: the activation of Wnt signaling pathways (Table 1 and Figure 2) phosphorylates and degrades of MYB protein [37], and BMP/TGF-beta and Wnt responsive OVOL1 antagonizes transcriptional activation of MYB by competing for target promoter binding [38].

Another novel transcriptional regulator for osteoblast differentiation we predicted is BAF57, which is the regulatory subunit SWI/SNF chromatin remodeling complex [39]. Multiple BAF57 target genes are directly related to osteoblast differentiation and function (Additional file 1: Supplementary Table 1). Indeed, SWI/SNF regulates osteoblast-specific transcription through chromatin structure modification [40]. BMP6 treatment may target SWI/SNF to nucleus though SMAD1 signal [41] or p38 MAPK pathway targets SWI-SNF chromatin-remodeling complex [42]. Interestingly, BAF57 positive target genes are up-regulated and negative targets down-regulated during BMP6 induction, which further confirms the involvement of BAF57 activity. The different timing of the positive and negative regulation likely suggests different dynamics of these actions.

Other interesting regulatory mechanisms are inferred based on the top ranking experimental sets (Table 4). Interferon beta (interferon alpha and gamma too, but not shown) positive target gene sets are down-regulated, which is consistent with Jak-STAT pathway from KEGG (Table 1). VEGF positive targets are down-regulated, likely because VEGF gene expression is down-regulated due to MYB inhibition [43]. IRS negative targets are down-regulated is consistent with activation of IGF signal, particularly up-regulation of IGF receptor binding proteins (Table 4 and Figure 5).

## Discussion and Conclusion

This is the first high throughput microarray study on BMP6 induced transcriptional program in human MSC. It covers the whole process from early to late stage osteoblast differentiation and mineralization. We conducted a comprehensive gene set analysis to identify relevant regulatory mechanisms and functional groups. We inferred a series of significant KEGG pathways, GO terms and experimental sets at different stages of BMP6 induction process. We not only showed which pathways or gene sets are significant, but also when and how they are involved in the osteoblast differentiation and mineralization. Different from common pathway analyses [13,14,16], our work further captures the interconnections among individual pathways or functional groups and integrate them into a whole system. Taken together, this work provides clearer mechanistic picture of osteoblast differentiation and function.

We inferred novel and coherent sets of regulatory mechanisms downstream of BMP6 signaling during osteoblast differentiation and mineralization. First, the same set of KEGG pathways are constantly involved in BMP6 induction. Their roles in osteogenic induction are clarified based on their perturbation patterns and connected to relevant discoveries in literature. These significant KEGG pathways are not separated but rather they work as a unified super regulatory system, and the pathway perturbation patterns we derived reflect a dynamic transmission process of the regulatory signal at transcriptional level along the super system. Second, a varying set of GO processes and functional groups are involved at different stage of BMP6 induced osteoblast differentiation and mineralization. These suggest novel yet plausible regulatory mechanisms, which are connected to but have not directly and explicitly introduced in literature works. Third, the most significant experimental sets suggest novel transcriptional regulators including MYB and BAF57, and regulatory pathways consistent with predictions based on KEGG and GO gene sets above.

Connections between KEGG pathways are evident as shown in the super regulatory network of pathways (Figure 4 and Table 2). Perturbations propagate along the super network at two levels: at protein level, the phosphorylation, binding, activation/inhibition events relay along pathways and transmit into interconnected pathways, as stated by KEGG graphs (Figure 3); at transcriptional level, gene expression perturbation propagates through auto-regulatory (feedback and feed forward) loops within pathways, and bridges into its neighbor pathways through the multiple component genes in common, as suggested by our pathway analysis results (Figure 4 and Table 2). Protein level transmission is faster, but transcriptional level transmission lasts longer hence ensure the long term biological effects. These longer lasting transcriptional effects are clearly evident in our data as the gene expression levels were perturbed long after the withdrawal of BMP6 treatment. The hard wired KEGG pathways and interconnections between them define how BMP6 signal triggers downstream programs (Figure 4).

There are also connections between BMP6 signal and the processes/groups represented by GO terms and experimental sets. For example, Notch signal and IGF signal are involved in the whole induction process (Figure 5a) like all significant KEGG pathways (Figure 2). It follows that these two signals should be also part of the super regulatory system and interconnected with multiple significant KEGG pathways of the network (Figure 4). This hypothesis is well supported by our data and literature: (1) Notch signal directly interacts with BMP signal. SMAD1 and NIC synergize to induce expression of HEY1 and other Notch targets [44-46]. Indeed up-regulation of HEY1 (and HEY2) requires continued BMP6 treatment by 96 hours (Additional file 1: Supplementary Figure 4c). Besides this binding-synergy at protein level, Notch ligand JAG1 expression is also up-regulated directly by BMP6 (Additional file 1: Supplementary Figure 4c). Notch also interacts with Wnt signal [47]. (2) Growth hormone (GH) signal [48] as part of cytokine-cytokine receptor interaction (KEGG pathway 04060) and Jak-STAT pathways (KEGG pathway 04630) are activated and by BMP signal (Figure 4) with GHR up-regulated (Additional file 1: Supplementary Figure 4a-b), GH signal up-regulates (IGF1 and IRS1, Additional file 1: Supplementary Figure 4d) and activates IGF signal in turn [48]. Connections between BMP signal and the two predicted transcriptional regulators, MYB and BAF57, are described above in the Results.

When examined together, we find a consistent picture emerging from the lists of significant KEGG pathways, GO terms, and experimental sets. For example, KEGG focal adhesion and ECM receptor interaction pathways (Table 1), GO homophilic cell adhesion (Table 3) and extracellular matrix structural constituent (not shown) groups consistently show the relevance of cell adhesion and extracellular matrix in osteoblast differentiation and mineralization. GO immune response groups (Table 3) echoes KEGG cytokine-cytokine receptor interaction pathway (Table 1). Similarly, significant experimental target genes sets (Table 4) closely reflected changes in the regulatory KEGG pathways (Table 1) or GO processes/groups (Table 3).

Interestingly, there are discrepancies among the clustering and the significant KEGG pathways, GO terms, and experimental sets. For example, Notch signaling is defined as both a KEGG pathway and a GO process. This KEGG pathway is not significant (not shown) but this GO process is (Figure 5). This discrepancy arises from two sources: (1) different definitions, i.e. KEGG pathways contain partially different set of genes from corresponding GO processes. While KEGG pathways tend to cover the whole homeostatic signal transmission systems even across multiple transcriptional cycles, GO usually covers one or multiple discrete steps or functional groups for a process. KEGG and GO definitions can be considered complementary and both provide valuable gene sets for our analysis. (2) GAGE [18] treats KEGG pathways and GO term gene sets differently: genes under a GO term are taken as a group coregulated towards a single direction, either all up or all down regulated, whereas genes in a KEGG pathway are frequently not coregulated and expression changes in both directions are counted. Timing discrepancies exist between experimental sets and corresponding KEGG pathways. For example, IFN positive target sets (only IFN beta shown) are not significant at 24-96 hours and MYB target set not at 96 hours (Figure 6) while Jak-STAT pathway and Wnt signaling pathway are significantly perturbed all the time (Figure 2). This can be explained by the fact that two-directional perturbation treatment for KEGG pathway does not account for direction or net effect of the perturbation, whether inhibited, activated or no overall effect. In the other hand, GO term analysis has no such issue, and IRS negative set and corresponding GO IGF receptor binding group are both significant all the time.

In this work, we took a systems approach in studying MSC differentiation. We combined experimental and computational work to reconstruct a unified picture of BMP6 signaling. The same set of experimental design and computational approach could be used to study other physiological and pathological processes, such as the differentiation of other cell types or tumorogenesis.

## Methods

### Cell culture and BMP6 osteogenic induction

Passage 5 human MSC (5 × 10^5^) were plated in 24-well dishes and cultured for 3 days. The cells were subsequently placed in serum free media supplemented with ITS for 24 hours. BMP6 was then added for the pre-defined time periods and then removed as shown in Figure 1. To remove BMP6, cells were rinsed 2 times before fresh media without BMP6 was added. Ascorbate and b-glycerolphosphate were added 4 days after the initiation of BMP treatment. Cells were harvested at the indicated time points. To quantifying mineralization, the plates were stained with Alizarin Red S [5] 14 days after the initiation of BMP treatment.

### Microarray experiment and analysis

Human MSC cells underwent osteogenic induction with BMP6 treatment for 0 hours, 8 hours, 24 hours, and 96 hours, which correspond to four phenotypic groups, i.e. control, preosteoblast (no mineralization), (sub-maximal) mineralization, and maximal mineralization at 14 days after the initiation of BMP treatment (18 days in total). Cells were harvested at 8 hours, 24 hours, 96 hours and 10 days for microarray profiling using Affymetrix U133 plus GeneChip^® ^platform. Assays were run in duplicate for a total of 20 arrays.

The raw data were processed by using FRAMS [49] with up-to-date probe set definition [44]. GAGE [18] was applied to infer the most differentially expressed pathways or gene sets between the BMP6 added or withdrawn samples under comparison at different time points (Figure 1). As is shown in Figure 1, the comparisons at 8, 24 and 96 hours were between two sample conditions, whereas comparison at 10 days was between the 24 and 96 hours BMP6 groups versus 0 and 8 hours BMP6 groups.

### Pathway analysis using GAGE

We use GAGE [18], Generally Applicable Gene set Enrichment, a novel method we developed for gene set/pathway analysis. The GAGE method is implemented in R in the "gage" package, available through Bioconductor at http://bioconductor.org/packages/release/bioc/html/gage.html. As the GAGE procedure has been described in detail in the original paper [18], here is a brief summary of the method.

Step 1: Gene sets separation. Gene sets are derived or collected from KEGG pathways [24], GO [50] and MSigDB [51] databases, as KEGG pathways, GO terms and experimental sets respectively. GAGE treats KEGG pathways differently from GO terms and experimental sets: member genes for a GO term or experimental set are taken as a group coregulated towards a single direction, either all up or all down regulated, whereas genes in a KEGG pathway are frequently not coregulated and expression changes in both directions are counted. We test for one-directional changes in GO terms because common function is frequently connected to coregulation and coexpression [52,53], which has also been extensively used in classical GO analysis tools [13,54,55]. Such treatment will miss some pathway-like GO terms which are significantly perturbed in two directions, but we intended to have GO analysis as a complementary work to the KEGG pathway analysis. Nonetheless, GAGE provides two-directional test options for all types of gene sets. For GO analysis, all terms are included without differentiating categories, i.e. Biological Process, Cellular Component, Molecular Function, and without considering the hierarchical organization of the ontology tree.

Step 2: One-on-one comparisons. Instead of comparing BMP6 treated samples vs controls as two groups, GAGE does one-on-one comparison between samples from the two groups at a time. For each one-on-one comparison, log based fold changes are calculated for all genes. GAGE conducts two-sample t-test on the average fold change in specific gene sets against that for the background of the whole set. This one-on-one comparison procedure is repeated for all potential experiment-control pairs.

Step 3: Summarization. For each gene set, GAGE derives a global P-value based on a meta test on the negative log sum of multiple P-values for this set from all one-on-one comparisons between experiments and controls.

### Overlap between significant pathways

Significant KEGG pathways may overlap in terms of perturbed member genes, which could suggest biologically meaningful connections between pathways. A gene is counted as perturbed when its absolute fold change is at least one standard deviation higher than the mean of all genes. The significance of overlap between pathways is inferred using hypergeometric test as following. Let *n *represent number of perturbed genes in a pathway, *N *number of all perturbed genes in a two-state comparison, *x *the overlap perturbed genes between pathways, pathways 1 and 2 are labeled by corresponding suffix. Then the chance to see *X *or more overlap perturbed genes between two significant pathways is:(3)

### Perturbation pattern visualization

For significant KEGG pathways, we generated graphs to visualize the dynamic expression perturbation at two levels: individual genes (Figure 3 and Additional file 2,3,4,5) and whole pathways (Figure 4). Gene expression level fold changes are standardized over the standard deviation of fold changes for all genes. The standardized fold changes for individual genes in KEGG pathways are visualized by using KEGGanim web tool [56] in Figure 3 and Additional file 2,3,4,5. We present an integrated network to show the connections between pathways and average expression perturbations for them in Figure 4. Significant KEGG pathways or closely related other pathways connected to them are represented by nodes. Connections between these pathways collected from KEGG database and graphs [24] are represented by arrows plus edges. The mean t-statistics from two-sample t-test from multiple one-on-one comparisons are plotted in pseudo heat colors as average perturbation magnitude for significant KEGG pathways.

## Authors' contributions

WL, MSF, KDH and PJW conceived and designed the study; WL conducted the computational analysis and result interpretation; MSF conducted the microarray experiment. WL and PJW drafted the manuscript. All authors read and approved the final manuscript.

## Supplementary Material

Additional file 1 **Supplementary Tables and Figures**. Click here for file

Additional file 2 **Supplementary Figure 1, animations of the dynamic gene expression perturbation patterns in TGF-beta signaling pathway**. Click here for file

Additional file 3 **Supplementary Figure 2, animations of the dynamic gene expression perturbation patterns in focal adhesion pathway**. Click here for file

Additional file 4 **Supplementary Figure 3, animations of the dynamic gene expression perturbation patterns in MAPK signaling pathway**. Click here for file

Additional file 5 **the input pathway expression data, i.e. standardized fold changes, used by KEGGanim web tool to generated the KEGG Pathway plots in Figure 3**. Click here for file

Additional file 6 **tables of significant KEGG pathways and GO groups inferred by GAGE at different time points**. Click here for file
