# Supplementary material for: Time series gene expression profiling and temporal regulatory pathway analysis of BMP6 induced osteoblast differentiation and mineralization
Source: BMC Syst Biol. 2011 May 23;5:82. doi: 10.1186/1752-0509-5-82 (PMC3126716; doi:10.1186/1752-0509-5-82)
Supplement: Additional file 1 — Supplementary Tables and Figures. [file 1752-0509-5-82-S1.DOC]

**Supplementary Tables and Figures**

**Supplementary Table 1**. Eleven BAF57 positive target genes (the ‘BAF57 up’ gene set) were evidently induced by 8 hours BMP6 treatment. A gene is counted as evidently induced with its fold change is one standard deviation higher than the mean for all genes. Eight of these genes are likely regulators for osteoblast differentiation based on their functional annotation.

| Gene | Relevant function |
| --- | --- |
| FZD1 | Wnt signaling |
| PRRX1 | Transcription factor |
| DIO2 | -- |
| AGC1 | ECM, osteoblast function |
| PRRX2 | Transcription factor |
| GHR | Osteoblast prolif./diff. |
| JAG1 | Notch signaling; mineralization |
| LBH | -- |
| BMPR2 | BMP signaling |
| LMCD1 | -- |
| SOCS2 | Insulin signaling, osteoblast function |

**Supplementary Figures 1-3**. Animations of the dynamic gene expression perturbation patterns of three representative KEGG pathways visualized using KEGGannim web tool. These Animated GIF files are not included in this supplementary document, but instead exist as individual Additional files (Additional file 2-4). The static perturbation patterns with these KEGG graphs are shown in Figure 3.


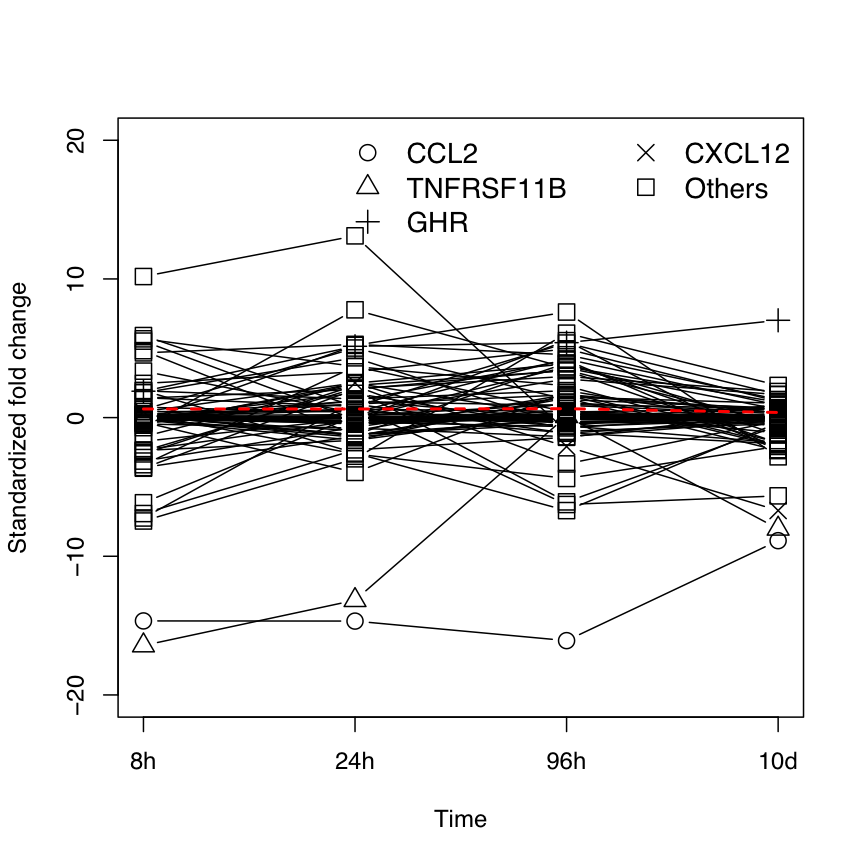

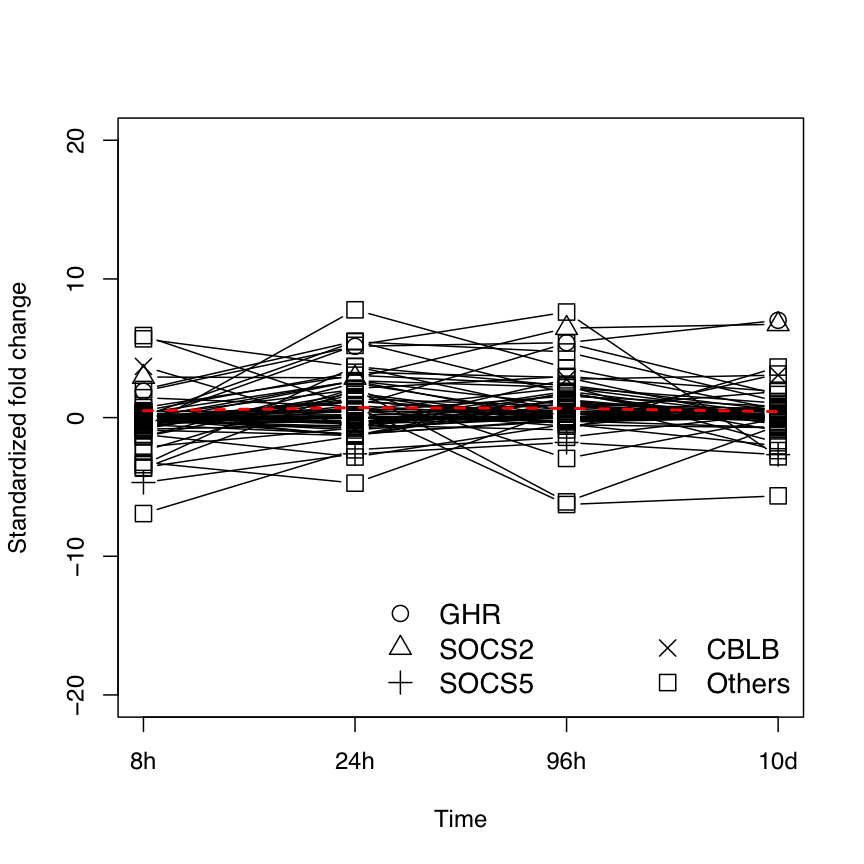

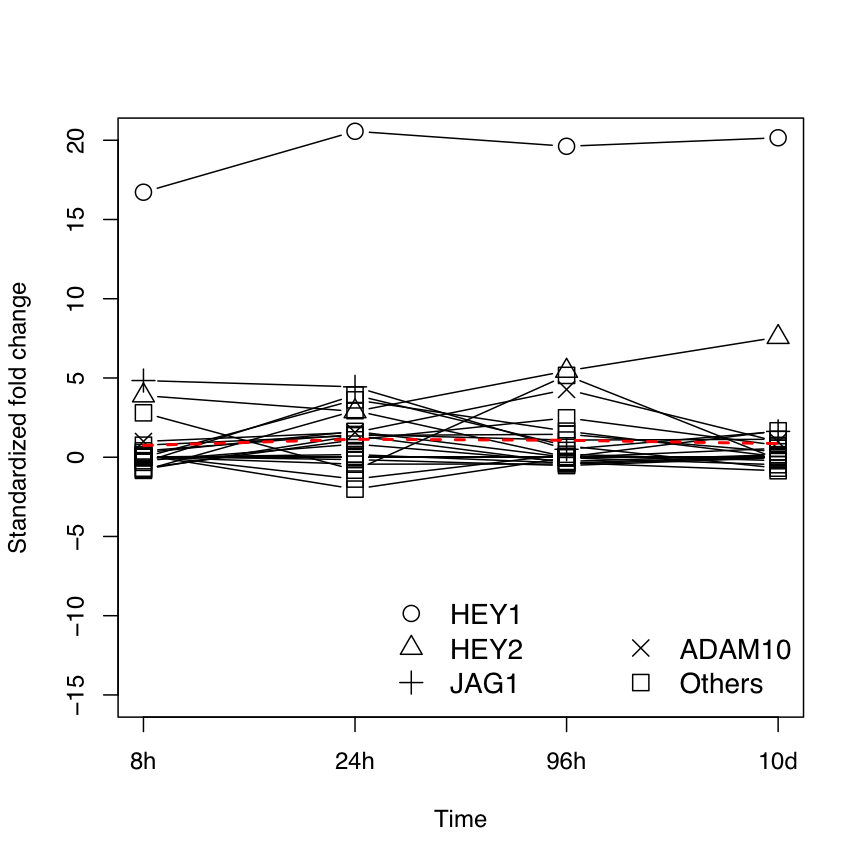

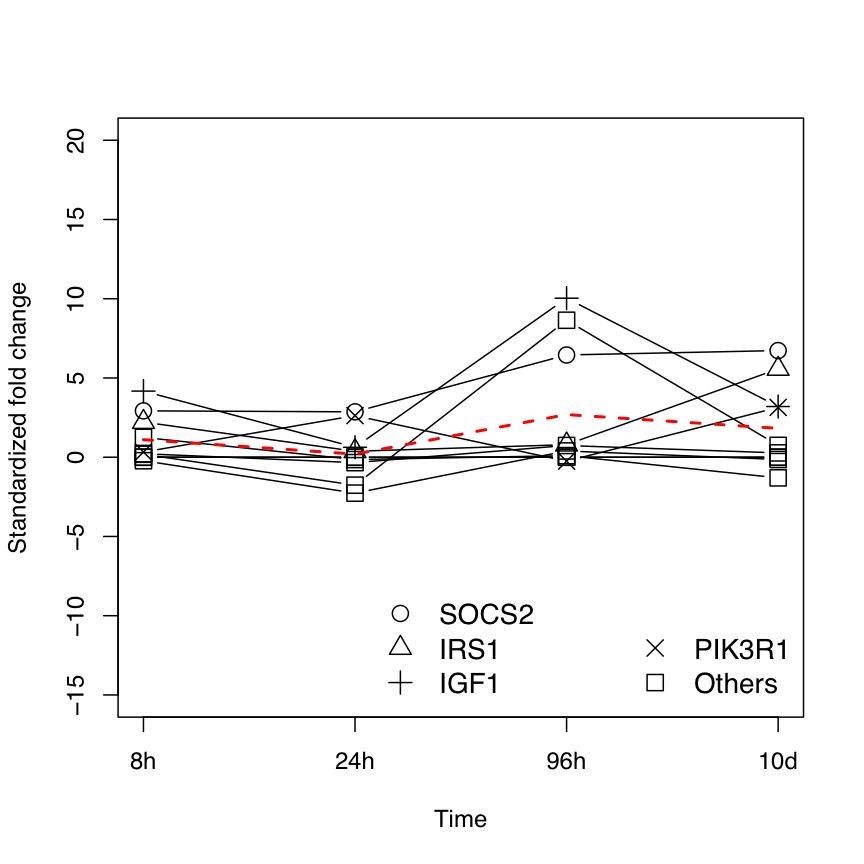

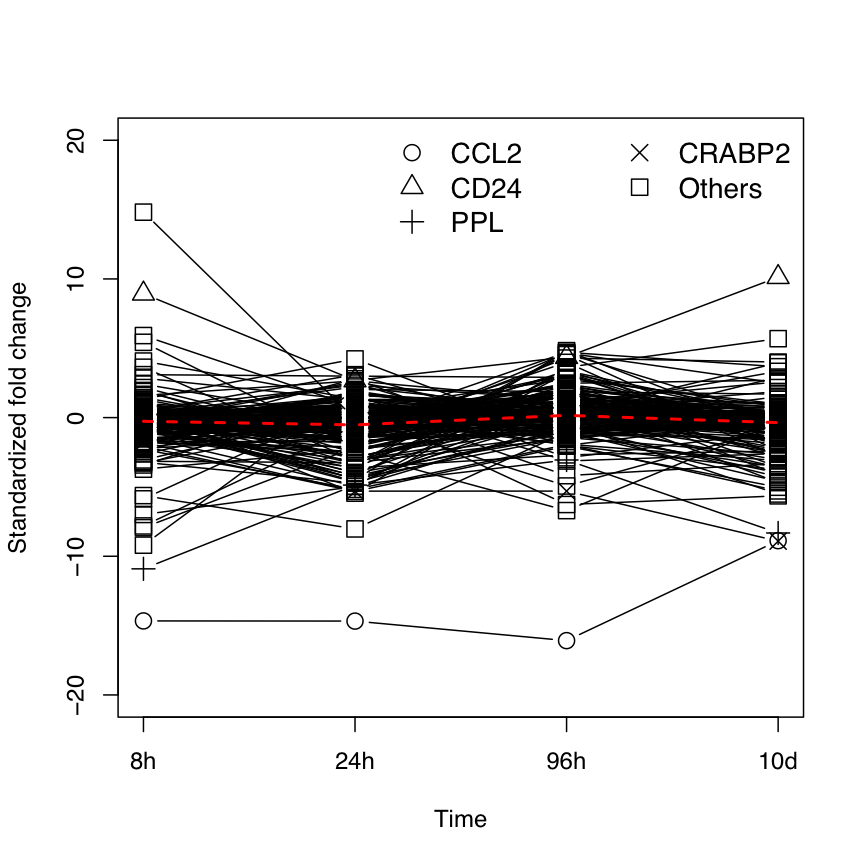

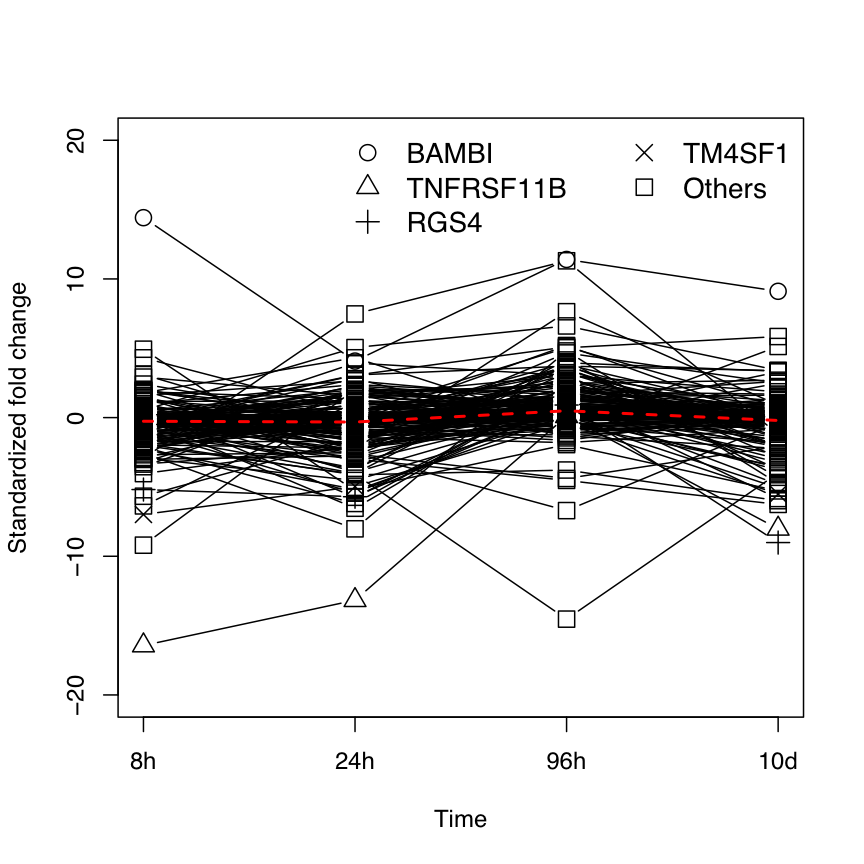


(a) Cytokine-cytokine receptor interaction (KEGG)

(b) Jak-STAT signaling pathway (KEGG)

(c) Notch signaling pathway (GO)

(d) Insulin-like growth factor receptor binding (GO)

**Supplementary Figure 4.** Individual gene expression perturbation patterns induced by BMP6 treatment in the representative significant gene sets. Log 2 based expression level fold changes induced by BMP6 treatment were standardized over the standard deviation for all genes. Top 4 most perturbed genes are labeled differently from the other genes. The red dashed line marks the mean for each gene set.

(e) MYB targets

(f) BAF57 down
