# Supplementary figures and images for: Time series gene expression profiling and temporal regulatory pathway analysis of BMP6 induced osteoblast differentiation and mineralization
Source: BMC Syst Biol. 2011 May 23;5:82. doi: 10.1186/1752-0509-5-82 (PMC3126716; doi:10.1186/1752-0509-5-82)

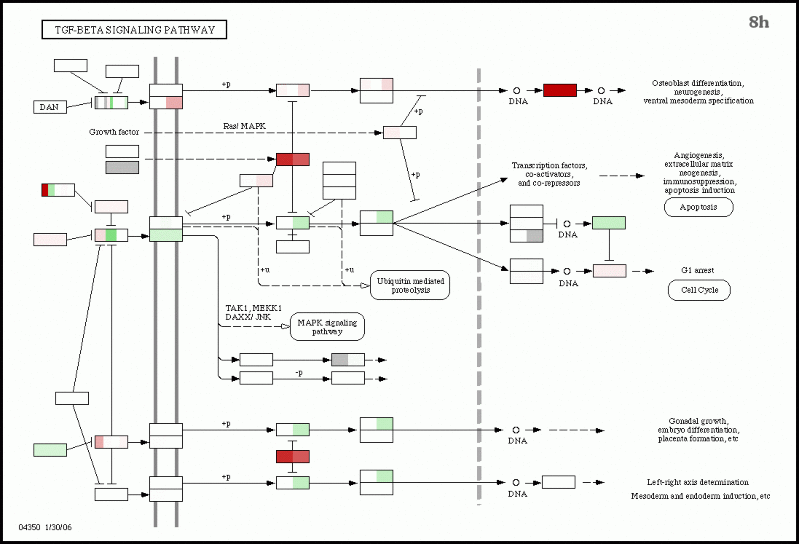

Supplement: Additional file 2 — Supplementary Figure 1, animations of the dynamic gene expression perturbation patterns in TGF-beta signaling pathway. [file 1752-0509-5-82-S2.GIF]

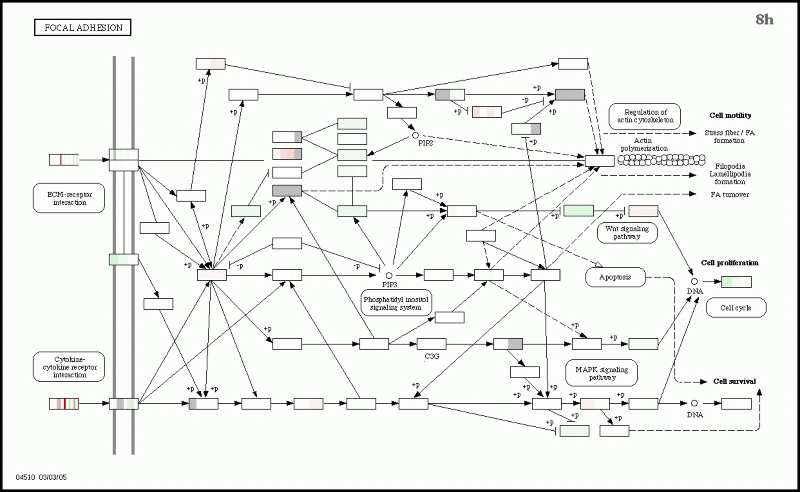

Supplement: Additional file 3 — Supplementary Figure 2, animations of the dynamic gene expression perturbation patterns in focal adhesion pathway. [file 1752-0509-5-82-S3.GIF]

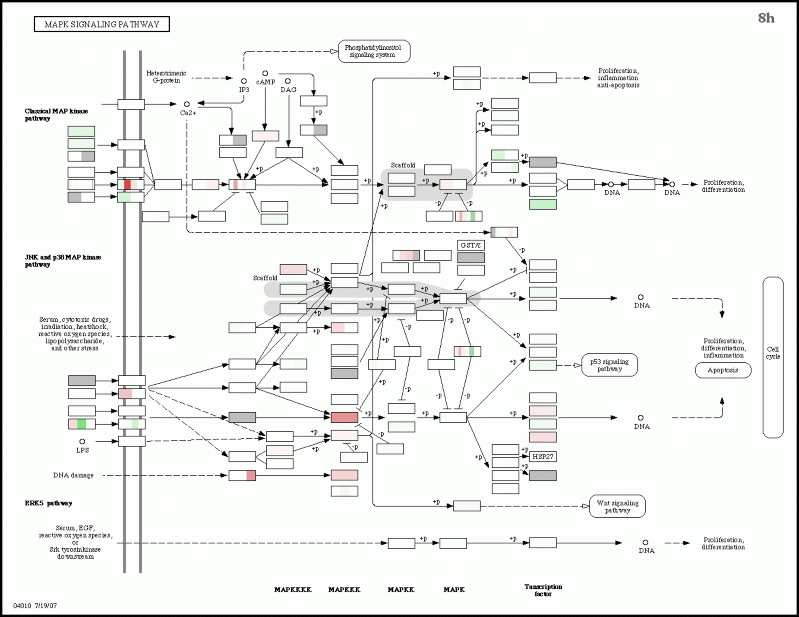

Supplement: Additional file 4 — Supplementary Figure 3, animations of the dynamic gene expression perturbation patterns in MAPK signaling pathway. [file 1752-0509-5-82-S4.GIF]
